# Broadband Optical Properties of Bi_2_Se_3_

**DOI:** 10.3390/nano13091460

**Published:** 2023-04-25

**Authors:** Georgy A. Ermolaev, Ivan S. Vyslanko, Andrey P. Tselin, Marwa A. El-Sayed, Mikhail K. Tatmyshevskiy, Aleksandr S. Slavich, Dmitry I. Yakubovsky, Mikhail S. Mironov, Arslan B. Mazitov, Amir Eghbali, Daria A. Panova, Roman I. Romanov, Andrey M. Markeev, Ivan A. Kruglov, Sergey M. Novikov, Andrey A. Vyshnevyy, Aleksey V. Arsenin, Valentyn S. Volkov

**Affiliations:** 1 Center for Photonics and 2D Materials, Moscow Institute of Physics and Technology, 9 Institutsky Lane, Dolgoprudny 141700, Russia; 2 Photonics and Quantum Materials Department, Skolkovo Institute of Science and Technology, 3 Nobel Str., Moscow 143026, Russia; 3 Department of Physics, Faculty of Science, Menoufia University, Shebin El-Koom 32511, Egypt; 4 Department of Solid State Physics and Nanosystems, National Research Nuclear University MEPhI (Moscow Engineering Physics Institute), 31 Kashirskoe Sh., Moscow 115409, Russia; 5 Center of Fundamental and Applied Research, Dukhov Research Institute of Automatics (VNIIA), 22 Suschevskaya Str., Moscow 127055, Russia; 6 Laboratory of Advanced Functional Materials, Yerevan State University, 1 Alek Manukyan Str., Yerevan 0025, Armenia

**Keywords:** transition metal dichalcogenides, optical constants, refractive index, topological insulators, nanophotonics, spectroscopic ellipsometry

## Abstract

Materials with high optical constants are of paramount importance for efficient light manipulation in nanophotonics applications. Recent advances in materials science have revealed that van der Waals (vdW) materials have large optical responses owing to strong in-plane covalent bonding and weak out-of-plane vdW interactions. However, the optical constants of vdW materials depend on numerous factors, e.g., synthesis and transfer method. Here, we demonstrate that in a broad spectral range (290–3300 nm) the refractive index *n* and the extinction coefficient *k* of Bi_2_Se_3_ are almost independent of synthesis technology, with only a ~10% difference in *n* and *k* between synthesis approaches, unlike other vdW materials, such as MoS_2_, which has a ~60% difference between synthesis approaches. As a practical demonstration, we showed, using the examples of biosensors and therapeutic nanoparticles, that this slight difference in optical constants results in reproducible efficiency in Bi_2_Se_3_-based photonic devices.

## 1. Introduction

High-refractive-index materials are the core of modern nanophotonics [1,2]. In particular, the real part n of the complex refractive index $\tilde{n}$ determines the photonic devices’ footprint, which scales as λ/n, where λ is a free space wavelength of light [3,4]. Moreover, the device performance is also highly dependent on n [5]. Hence, even a minor increase in the refractive index has a significant impact on nanophotonics [1]. However, classical high-refractive-index materials (TiO_2_, GaP, Si, and Ge) offer a limited range of available refractive indices, and these typically fall below 4 [2,6,7,8]. It is thus necessary to search for new optical materials with large optical responses [1].

**Figure A1:**
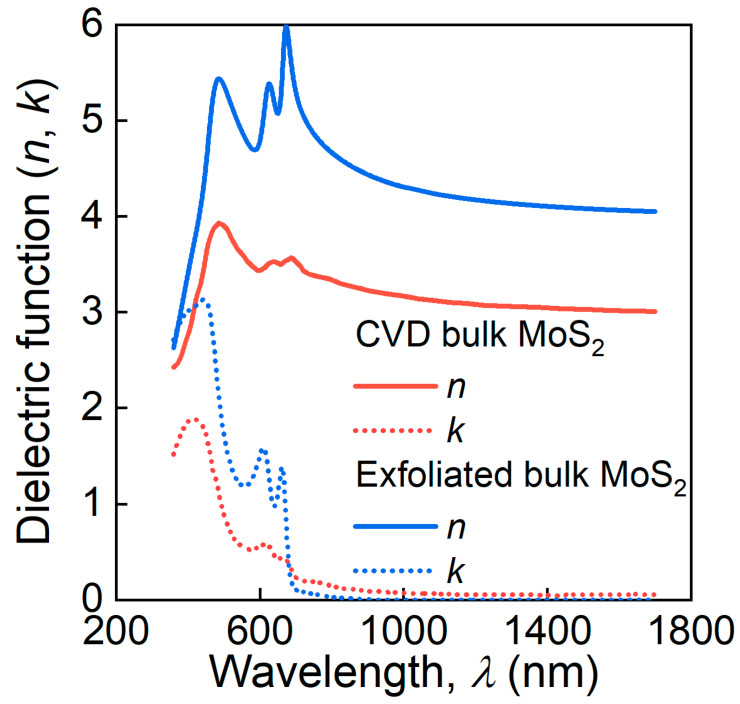
Comparison of dielectric functions of chemical vapor deposition-grown [27] and exfoliated [12] MoS_2_.

The appearance of graphene and other two-dimensional (2D) and layered materials [9,10,11] has led to the development of a novel materials platform, usually referred to as van der Waals (vdW) materials [12,13]. To date, there are more than 5000 potential vdW crystal structures which provide diverse optical responses [14] and can be employed together with or fully independently of bulk materials. One of the most promising is a family of transition metal dichalcogenides (TMDCs), which includes MoS_2_, WS_2_, MoSe_2_, and WSe_2_ [12,15,16,17,18]. Due to their pronounced in-plane excitonic effects, they offer previously unimaginable optical constants with in-plane refractive indices exceeding 4 [12,19,20]. As a result, numerous studies consider vdW materials to be essential building blocks for next-generation nanophotonics [1,4,12,21]. However, industrial implementation requires standardized optical responses, i.e., sample-to-sample variations in optical properties should be very low. By contrast, the optical constants of vdW materials depend on numerous factors [22,23,24,25,26], among which the most influential are the dielectric environment and the synthesis method. For example, recent research [23] has demonstrated that optical constants vary dramatically among differently synthesized samples of monolayer MoS_2_. At λ= 750 nm, the refractive indices n of exfoliated, epitaxial, and chemical vapor deposition-grown MoS_2_ are equal to 3.16, 4.02, and 5.16, respectively [23]. This is a more than 60% variation in optical constants, which greatly impedes the commercial viability of TMDCs. Furthermore, the considerable differences between the optical constants of differently synthesized samples is not only problematic for 2D materials, but for bulk crystals too, as can be seen from the comparison of dielectric functions of chemical vapor deposition-grown [27] and exfoliated [12] MoS_2_ (Figure A1). Therefore, vdW materials with synthesis-independent optical properties are in high demand.

To address this issue, we focused on vdW topological insulators such as Bi_2_Se_3_, Bi_2_S_3_, Bi_2_Te_3_, and Sb_2_Te_3_ [28,29,30,31,32,33,34]. Their topological nature has attracted renewed interest in their electronic and optical properties [32,35,36,37,38,39,40,41]. More importantly, they demonstrate an even stronger optical response than TMDCs with a refractive index above 5 [32,40,42,43]. This enables, for example, the realization of pronounced Mie resonances with high-order multipoles [32]. Therefore, their optical constants are of great interest. Finally, one might assume that the optical responses of vdW topological insulators is less dependent on the synthesis process than those of TMDCs because of the topological protection of their topological surface states [44].

In this study, we investigated the dependence of the broadband optical properties of Bi_2_Se_3_, which is a typical vdW topological insulator, on the synthesis method. The comparison of spectroscopic ellipsometry results and first-principle computations together with the literature database reveal that Bi_2_Se_3_ has synthesis-independent optical constants. Additionally, we determined the broadband (290–3300 nm) refractive index and extinction coefficient. Our findings show that Bi_2_Se_3_ has a high refractive index (n~5) and extinction coefficient (k> 0.1) for a wide spectral interval, which makes Bi_2_Se_3_ a promising material for a wide range of photonic applications.

## 2. Materials and Methods

### 2.1. Materials

Full area coverage Bi_2_Se_3_ thin film was purchased from SixCarbon (6CarbonTechnology, Shenzhen, China), where the sample was synthesized via the chemical vapor deposition (CVD) method on a silicon substrate with silicon dioxide.

### 2.2. Raman Characterization

A Horiba LabRAM HR Evolution confocal scanning Raman microscope (Horiba Ltd., Kyoto, Japan) was employed for the acquisition of the Raman spectra of the Bi_2_Se_3_. All measurements were carried out under linearly polarized excitation at free space wavelengths of 532 nm and 632.8 nm with a 1800 lines/mm diffraction grating and a 100× objective with a numerical aperture (NA) of 0.90. The spot diameter was approximately 0.9 µm. The Raman spectra were recorded with an integration time of 10 s.

### 2.3. Spectroscopic Ellipsometry Characterization

A variable-angle spectroscopic ellipsometer (VASE, J.A. Woollam Co., Lincoln, NE, USA) was utilized to measure the broadband optical constants of the Bi_2_Se_3_. The measurements were performed at multiple incident angles from 50° to 70° in 5° steps, and over a broad spectral interval from 290 nm to 3300 nm. For the ellipsometry spectra analysis, we employed WVASE software, provided by the producer. We described the Bi_2_Se_3_ sample using a four-layer model: roughness layer, Bi_2_Se_3_ layer, silicon dioxide (SiO_2_), and silicon (Si) substrate. The thicknesses of the layers were 21.7 nm (for the roughness layer), 44.4 nm (for the Bi_2_Se_3_ layer), 265.7 nm (for the SiO_2_ layer), and a semi-infinite layer for the silicon. In order to account for the surface roughness, we followed a standard approach [45] which involved introducing the Bruggeman effective medium approximation layer with equal fractions of air and investigating the Bi_2_Se_3_ material. In addition, despite the uniaxial anisotropy of the Bi_2_Se_3_ [12], we used an isotropic model for the Bi_2_Se_3_ since ellipsometry is almost insensitive to the out-of-plane component because of the high in-plane refractive index, which significantly decreases the interaction between the light electric field and the out-of-plane dielectric function of the film.

### 2.4. Reflectance Measurements

The reflectance spectrum (λ= 450–950 nm) of the Bi_2_Se_3_ sample was measured using a Biolam M-1 optical microscope (LOMO, Saint Petersburg, Russia) equipped with a 24 V, 100 W halogen light source and a QE65000 spectrometer (Ocean Optics, Dunedin, FL, USA).

### 2.5. First-Principle Calculations

The optical constants of the Bi_2_Se_3_ were calculated using the GW@DFT approach implemented in the VASP package [46]. First, the atomic positions of the crystal (a=b=0.4143 nm and c=2.8636 nm) [47] were relaxed until the converged interatomic forces were less than 10^−2^ eV/nm, and the unit cell was kept fixed. Next, we obtained ground-state one-electron wavefunctions using the density functional theory (DFT) and used them to initialize the GW routines. Finally, we calculated the imaginary and real parts of the frequency-dependent dielectric function within the GW approximation and derived the refractive indices and extinction coefficients of the material. The cutoff energy for the plane-wave basis set was 500 eV, while the first Brillouin zone was sampled with a Γ-centered 18 × 18 × 3 grid. The exchange correlation effects were described with a generalized gradient approximation (Perdew–Burke–Ernzerhof functional) [48], and the behavior of the wavefunctions in the core region was reconstructed with a projector augmented wave pseudopotentials [49].

### 2.6. Optical Visualization

The optical images of the Bi_2_Se_3_ samples were recorded using an optical microscope (Nikon LV150L, Tokyo, Japan).

### 2.7. Atomic-Force Microscopy

The surface topography of the Bi_2_Se_3_ films was examined using an atomic force microscope (NT-MDT Ntegra) operated in a semi-contact mode. AFM scanning was performed in air using HA_NC ETALON silicon tips (TipsNano, Tallinn, Estonia) with a tip-tapping resonant frequency of around 140 kHz and a spring constant of 3.5 N/m. The quantitative analysis was carried out using Gwyddion software (www.gwyddion.net; accessed on 1 January 2022).

### 2.8. X-ray Photoelectron Spectroscopy

The chemical state and composition were analyzed via X-ray photoelectron spectroscopy (XPS) using a Theta Probe spectrometer under high-vacuum conditions (base pressure < 2 × 10^−9^ mbar) with a monochromatic Al-Kα X-ray source (1486.6 eV). The photoelectron spectra were acquired using the fixed analyzer transmission (FAT) mode with 50 eV pass energy. The spectrometer energy scale was calibrated on the Au4f_7/2_ line (84.0 eV). The XPS spectra were acquired using charge-compensation under the pressure of ~10^−7^ mbar to avoid sample charging. For the elemental composition XPS analysis, Scofield’s Factors were employed in the calculations.

## 3. Results and Discussion

### 3.1. Sample Characterization

**Figure 1:**
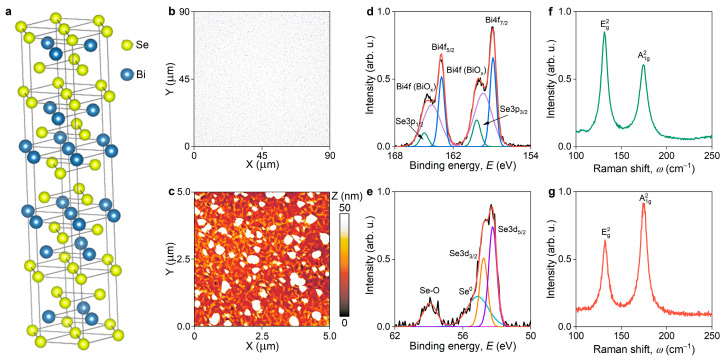
Bi_2_Se_3_ sample characterization. (**a**) Crystal structure of Bi_2_Se_3_. (**b**) Optical image of Bi_2_Se_3_ sample. (**c**) AFM color map of Bi_2_Se_3_ sample. (**d**,**e**) XPS spectra of Bi_2_Se_3_ with several Bi and Se peaks: Se3p_1/2_ (165.0 eV); Bi4f (BiO_x_) (164.2 eV); Bi4f_5/2_ (163.2 eV); Bi4f (BiO_x_) (158.9 eV); Bi4f_7/2_ (157.9 eV); Se3p_3/2_ (159.6 eV); Se-O (58.9 eV); Se^0^ (54.7 eV); Se3d_3/2_ (54.2 eV); Se3d_5/2_ (53.4 eV). Black, red, green, purple, blue, orange, violet, and cyan colors label experimental, total, Se3p_1/2_ or Se3p_3/2_, Bi4f (BiO_x_), Bi4f_5/2_ or Bi4f_7/2_, Se3d_3/2_, Se3d_5/2_, and Se^0^ XPS signals, respectively. Raman spectra of Bi_2_Se_3_ for (**f**) λ= 532 nm and (**g**) λ= 632.8 nm.

**Figure A2:**
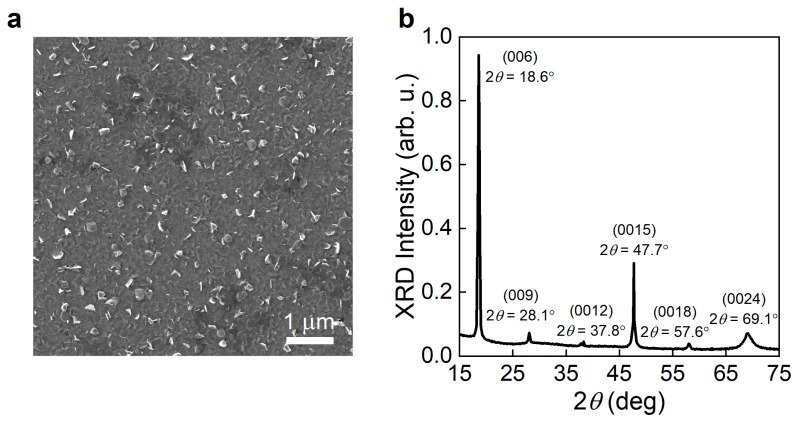
Additional structural and morphological study of Bi_2_Se_3_. (**a**) Scanning electron microscopy image of Bi_2_Se_3_ surface. (**b**) X-ray diffraction pattern of Bi_2_Se_3_ with pronounced peaks which correspond to the (006), (009), (0012), (0015), (0018), and (0024) crystallographic planes of Bi_2_Se_3_.

Bi_2_Se_3_ has a rhombohedral phase crystal structure with quintuple layers (Figure 1a). Our Bi_2_Se_3_ thin film was prepared using the chemical vapor deposition (CVD) method and, therefore, had a uniform substrate coverage, confirmed by optical microscopy (Figure 1b). From the optical image in Figure 1b, one can notice that the synthesized sample has a roughness, similar to other Bi_2_Se_3_ samples grown using CVD [50,51]. In order to obtain a qualitative estimate of the roughness, we measured the sample surface with an atomic-force microscope (Figure 1c). The AFM image yielded a root mean square roughness of 26 nm. After that, we verified the stoichiometry of the samples using X-ray photoemission spectroscopy (XPS) (Figure 1d,e). The XPS signal in Figure 1d,e shows a rich spectrum with Bi- and Se-related peaks, and a quantitative XPS analysis based on Bi4f and Se3d showed that the stoichiometry of the film reached ~43%:57%, close to the expected 40%:60% (2 Bi: 3 Se) [52,53,54,55]. According to the previous studies of Bi_2_Se_3_, the peak Se^0^ (Figure 1e) indicates elemental selenium (Se) [55,56,57]. Additionally, we performed Raman spectroscopy (Figure 1f,g) at two excitation wavelengths: 532 nm (Figure 1f) and 632.8 nm (Figure 1g). Both spectra have two pronounced Raman peaks at 131.3 cm^−1^ and 174.3 cm^−1^ for λ= 532 nm and 131.9 cm^−1^ and 174.8 cm^−1^ for λ= 632.8 nm (Figure 1f,g). A comparison with Bi_2_Se_3_ data from the literature allowed us to assign the first peak with the $E_{g}^{2}$ phonon mode and the second peak with the $A_{1g}^{2}$ phonon mode, and their positions are very close to those reported for Bi_2_Se_3_ ($A_{1g}^{2}\;\sim$131 cm^−1^ and $E_{g}^{2}\;\sim$174 cm^−1^) with a thickness above 20 nm [58]. In addition, we performed scanning electron microscopy (SEM) and X-ray diffraction (XRD), as is shown in Figure A2. The SEM image in Figure A2a confirms the morphology of our sample surface as observed using atomic-force microscopy (Figure 1c), and the XRD pattern (Figure A2b) gives additional verification of Bi_2_Se_3_ crystal structure: the XRD peaks at around 9.3°, 18.6°, 28.1°, 37.8°, 47.7°, 57.6°, and 69.1° correspond to the (003), (006), (009), (0012), (0015), (0018), and (0021) crystallographic planes of the Bi_2_Se_3_, respectively [59]. In addition, we would like to note that the XPS spectra (Figure 1d,e) demonstrate the slight oxidation of the sample. However, other techniques, such as Raman spectroscopy (Figure 1f,g) and XRD (Figure A2b), show only the presence of Bi_2_Se_3_, which additionally confirms that the sample was only slightly oxidized. Hence, this preliminary sample characterization confirmed that our sample was Bi_2_Se_3_ and gives additional information about its roughness, which should be about 26 nm, and its thickness, which should be more than 20 nm.

### 3.2. Spectrocopic Ellipsometry of Bi_2_Se_3_

**Figure 2:**
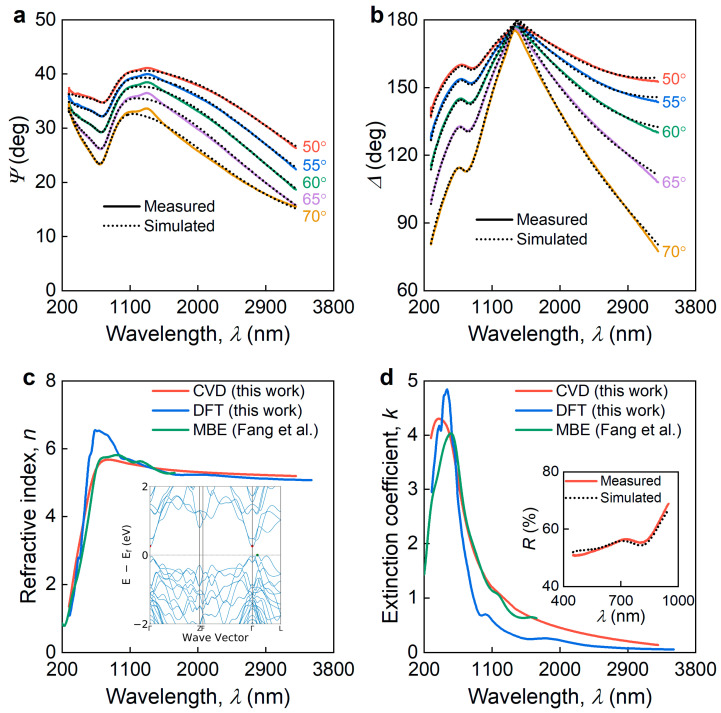
Variable-angle spectroscopic ellipsometry of Bi_2_Se_3_. Ellipsometry spectra of Bi_2_Se_3_ (**a**) Ψ and (**b**) Δ. Solid and dashed lines denote the experimental and calculated optical model data. (**c**) Refractive index and (**d**) extinction coefficient of Bi_2_Se_3_ for differently synthesized samples: chemical vapor deposition (CVD); density functional theory (DFT) calculations attributed to exfoliation; and molecular beam epitaxy (MBE), adopted from [40]. The inset in panel (**c**) is the calculated band structure of Bi_2_Se_3_. The inset in panel (**d**) shows a comparison between the experimental reflectance spectra of Bi_2_Se_3_ and the simulated one. Tabulated optical constants of Bi_2_Se_3_ are collected in Table A1.

To obtain the broadband optical properties of the Bi_2_Se_3_, we measured the spectroscopic ellipsometry of our sample at several incident angles (θ= 50–70°) in a broad wavelength range (λ= 290–3300 nm). The resulting spectra of ellipsometric parameters Ψ and Δ are plotted in Figure 2a,b. Since ellipsometry is a very accurate technique [60] that “feels” a system’s nonidealities, we included in the optical model an effective medium approximation (EMA) layer [45,61] on top of the Bi_2_Se_3_ film to account for the surface roughness (Figure 1b,c). We also assumed a negligible optical response from the Bi_2_Se_3_ oxide and the surface conductive layer, and therefore did not include it in the optical model. Our ellipsometry analysis started with a point-by-point conversion approach [62]. We then used the results from the first step for the Lorentz oscillator description of the optical constants of the Bi_2_Se_3_ (Figure 2c,d). We would like to note that unlike those of other semiconducting layered materials, the Bi_2_Se_3_ optical response is better described via Lorentz oscillators than via Tauc-Lorentz oscillators [63] since Bi_2_Se_3_ is a narrow bandgap ($E_{g}\;\approx$ 0.3 eV) semiconductor [34]. In other words, the lowest energy incident photon has 0.376 eV energy, which is much larger than the bandgap $E_{g}=$ 0.3 eV of Bi_2_Se_3_. We also confirmed this bandgap value with band structure computations (the inset in Figure 2c) using density functional theory. Furthermore, to validate the Bi_2_Se_3_ optical constants in Figure 2c,d and their predictive capabilities, we recorded the reflectance spectrum of our sample (the inset in Figure 2d) and compared it with the transfer matrix calculations [64] based on the refractive indices and extinction coefficients presented in Figure 2c,d. Therefore, our assumptions concerning a negligible optical response from the oxide layer and the EMA approach for roughness are valid because they were double-checked against the first-principle calculations and reflectance measurements, and there was good agreement between the AFM roughness of 26 nm and the effective ellipsometry roughness of 21.7 nm.

In addition to this, we compared the resulting dielectric function of our CVD-grown sample with the first-principle calculations and dielectric function of molecular beam epitaxy (MBE)-grown Bi_2_Se_3_, reported by Fang and colleagues [40]. Interestingly, our recent publications [12,65] have shown that DFT dielectric function coincides with the optical response of monocrystals (an almost perfect match for *n* and a qualitative match with *k*), which in the case of layered materials are usually prepared using the exfoliation technique [66]. Hence, we can safely assume that DFT optical constants correspond with exfoliated Bi_2_Se_3_. In this case, the perfect match between the CVD-grown, exfoliated, and MBE-grown Bi_2_Se_3_ (Figure 2c,d) implies that the Bi_2_Se_3_ optical response is almost synthesis-invariant, unlike those of other vdW materials [23]. This property makes Bi_2_Se_3_ a promising vdW material for commercial use because its optical properties are reproducible.

In addition to CVD and MBE technologies, Bi_2_Se_3_ can also be synthesized using numerous other methods, e.g., solvothermal [67] and sonochemical [68] methods and mechanical exfoliation [69]. Unfortunately, for most synthesis methods, it is hard to find optical constants for comparison because researchers now focus primarily on the electronic properties of topological states rather than the optics of Bi_2_Se_3_. Nevertheless, the CVD and MBE methods are the most popular and well-developed for synthesizing two-dimensional and layered materials and, therefore, the most important for the scientific community. Additionally, we provide first-principle computations, which give optical constants close to those of the exfoliated samples [12]. Hence, we can conclude that first-principle computations yield optical constants for exfoliated Bi_2_Se_3_, expanding our comparison to the three synthesis methods (CVD, MBE, and exfoliated) and confirming the synthesis-independent optical response of Bi_2_Se_3_.

### 3.3. Applications of Bi_2_Se_3_

To demonstrate the invariant performance of Bi_2_Se_3_-based photonic devices, we chose two applications: a surface plasmon resonance (SPR) biosensor [70] and the heating of nanoparticles for cancer treatment [21]. Performance-invariance is imperative for the reliable industrial implementation of Bi_2_Se_3_.

**Figure 3:**
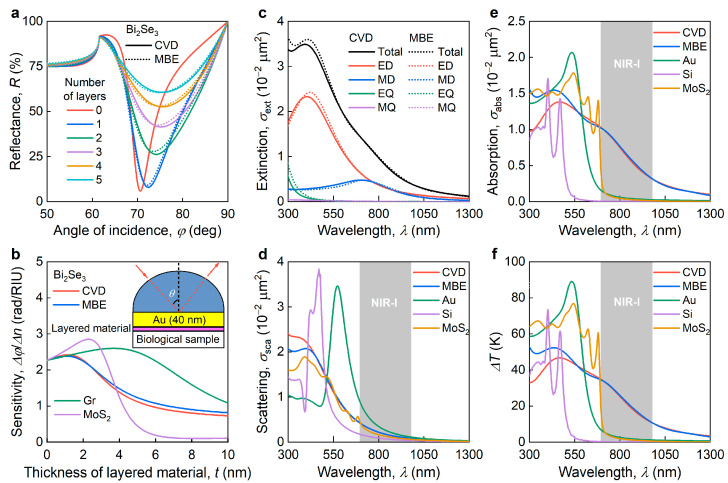
Photonic applications of Bi_2_Se_3_. (**a**) The reflectance of the surface plasmon resonance (SPR) sensor based on a SiO_2_/Au (40 nm) chip with CVD and MBE-grown Bi_2_Se_3_. (**b**) The dependence of SPR sensor angular sensitivity on the thickness of Bi_2_Se_3_ layers. The inset is a schematic representation of an SPR sensor. For comparison, we also added the Gr and MoS_2_ performance. (**c**) The multipole decomposition of the extinction spectrum of a single Bi_2_Se_3_ nanosphere with a diameter *d* of 100 nm. (**d**) Extinction and (**e**) absorption cross-section of nanoparticles with diameters d of 100 nm for CVD and MBE-grown Bi_2_Se_3_. (**f**) Spectral dependence of the heating of nanoparticles with diameters d of 100 nm for CVD and MBE-grown Bi_2_Se_3_. The gray regions in panels c–f show spectral therapeutic region NIR-I (700–980 nm). For comparison, we included the performance of Au, Si, and MoS_2_ NPs.

**Figure A3:**
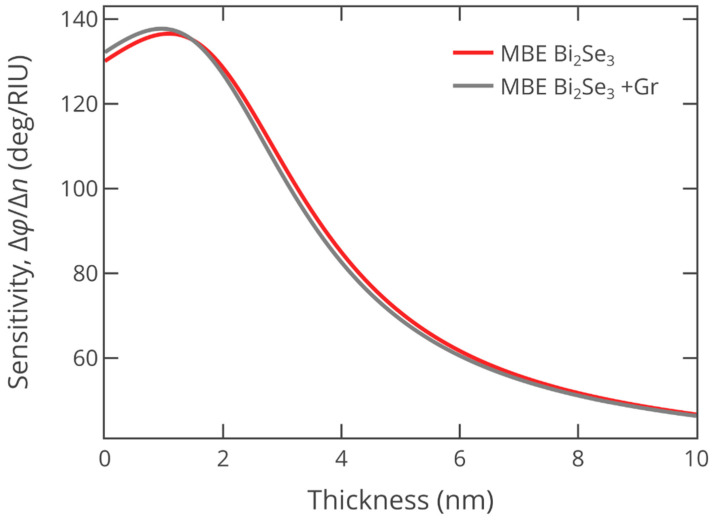
Angular optical sensitivity of the SPR biosensor as a function of the thickness of the auxiliary Bi_2_Se_3_ layer. Calculations were performed neglecting the optical conductivity contribution from the topological surface states (red line) and accounting for this by adding a monolayer of graphene (gray line).

We commence with a Bi_2_Se_3_-based SPR biosensor. In a common approach [70] to SPR-sensitivity enhancement, one usually deposits vdW materials on top of gold (or other plasmonic material) in a biosensor using the Kretschmann scheme (the inset in Figure 3b) [71]. The benefit of the added vdW material is twofold: (i) it increases the sensitivity of the biosensor; (ii) it enhances the immobilization efficiency of the detected molecules. Since Bi_2_Se_3_ is a topological insulator, one might expect that Bi_2_Se_3_ could also give a plasmonic response. This expectation is correct, and plasmonic modes in Bi_2_Se_3_ were observed in the THz range [39,72]. Above the bandgap, the optical response from the topological states is combined with interband transitions in bulk material, with the weight of the former decreasing with the increase in the thickness of the material [40,73]. At the same time, topological insulators can support guided surface electromagnetic waves, provided that the real part of the permittivity is negative [31,74]. In the case of Bi_2_Se_3_, at a standard SPR wavelength of *λ* = 635 nm (*E* = 1.953 eV), the real part of the dielectric permittivity is positive, and thus we do not foresee its application as a replacement for plasmonic metal. Assuming that Bi_2_Se_3_ is an auxiliary layer, the optical constants in Figure 2c,d allow the estimation of the sensitivity enhancement. Using the transfer matrix calculations, we determined the dependence of the reflection coefficient on the angle of incidence (Figure 3a) and the biosensor sensitivity (Figure 3b) for the CVD and MBE-grown Bi_2_Se_3_. The close characteristics (Figure 3a,b) of the biosensors for both the CVD and MBE Bi_2_Se_3_ show that the device performance remains almost independent of the choice of synthesis method. During the calculations, we neglected the contribution of the topological states to the optical response (see Figure A3 for the estimation of the error introduced by this oversight).

Given the recent success of the fabrication of nanospheres from vdW materials [21,75], we also considered Bi_2_Se_3_ nanospheres for efficient heating in the therapeutic window, known as NIR-I (700–980 nm) [21]. To demonstrate the efficiency invariance of Bi_2_Se_3_ for the optical response of nanoparticles, we employed the Mie theory [76] to calculate the multipole decomposition of the extinction spectrum (Figure 3c), scattering (Figure 3d), and absorption (Figure 3e) cross-sections for a nanosphere with a standard diameter of d= 100 nm in a water environment using the dielectric function of CVD and MBE-grown Bi_2_Se_3_. Using these cross-sections, we estimated the spectral dependence of the heating of Bi_2_Se_3_ nanoparticles under constant laser irradiation ($I_{0}=$ 3.2·10^5^ W/m^2^). Like the biosensor, the heating efficiency of the Bi_2_Se_3_ nanospheres was very close for CVD and MBE-grown Bi_2_Se_3_, especially in the practically important NIR-I spectral region. Thus, the synthesis-independent optical constants of Bi_2_Se_3_ lead to the synthesis-independent performance of Bi_2_Se_3_ optical devices.

In addition, it is worth comparing the performance of Bi_2_Se_3_-based devices with the performance of devices made from other materials. For the SPR comparison, we included the performance of graphene (Gr) [62] and MoS_2_ [77] in Figure 2b. This shows that despite the enormously high refractive index of Bi_2_Se_3_ (*n*~5.3) at a standard SPR wavelength of *λ* = 635 nm, Bi_2_Se_3_ demonstrates slightly less SPR sensitivity than graphene and MoS_2_ owing to its strong optical absorption (*k*~3.3). In addition, large optical constants make Bi_2_Se_3_ a suitable material for the heating of NPs for cancer treatment. Indeed, the comparison of Bi_2_Se_3_ with traditional materials such as Au [78], Si [79], and MoS_2_ [21] in the therapeutic window NIR-I reveals a more than tenfold enhancement in heating efficiency (Figure 3f). Therefore, Bi_2_Se_3_ is a promising material for absorbing and heating photonic applications thanks to its extraordinarily high optical response.

## 4. Conclusions

In summary, we have reported the broadband (290–3300 nm) optical properties of Bi_2_Se_3_, a typical representative of van der Waals (vdW) topological insulators. Our study shows that Bi_2_Se_3_ has ultrawide absorption, with an extinction coefficient above 0.1, and an enormously large dielectric response, with a refractive index above 5. This was unambiguously verified using theoretical computations within the density functional theory framework and reflectance spectroscopy. More importantly, we found that Bi_2_Se_3_ optical constants are synthesis-invariant, which is highly desirable for optical engineering. As a result, we envision Bi_2_Se_3_ as an essential material in the next generation of nanophotonic nanostructures, useful in countless applications, including biosensing [70], theranostics [21], photodetection [80], light focusing [81], and superabsorbers [82].

## Data Availability

The data presented in this study are available upon reasonable request from the corresponding author.

## Appendix A

**Table A1:** Tabulated optical constants for Bi_2_Se_3_ from Figure 2c,d.

*λ* (nm)	*n*	*k*	*ε*_1_	*ε*_2_
300	1.4835	4.0190	−13.9516	11.9244
350	2.1871	4.2554	−13.3252	18.6138
400	2.8587	4.3035	−10.3475	24.6049
450	3.4575	4.2646	−6.2330	29.4897
500	4.0442	4.1643	−0.9861	33.6823
532	4.4169	4.0377	3.2065	35.6679
550	4.6163	3.9393	5.7915	36.3699
600	5.0870	3.5798	13.0626	36.4213
633	5.3118	3.3042	17.2968	35.1024
650	5.4007	3.1606	19.1778	34.1386
700	5.5739	2.7596	23.4533	30.7635
750	5.6530	2.4133	26.1321	27.2847
780	5.6724	2.2346	27.1822	25.3509
800	5.6776	2.1268	27.7121	24.1507
850	5.6735	1.8926	28.6069	21.4748
900	5.6552	1.7003	29.0901	19.2311
1064	5.5695	1.2630	29.4246	14.0680
1200	5.5128	1.0702	29.2461	11.7991
1500	5.4053	0.7272	28.6883	7.8610
1800	5.3423	0.5421	28.2465	5.7919
2100	5.2989	0.4154	27.9057	4.4021
2400	5.2660	0.3213	27.6271	3.3839
2700	5.2390	0.2477	27.3855	2.5952
3000	5.2155	0.1880	27.1665	1.9612
3300	5.1943	0.1385	26.9619	1.4385
